# Crosstalk between Akt signaling and cold shock proteins in mediating invasive cell phenotypes

**DOI:** 10.18632/oncotarget.24886

**Published:** 2018-04-10

**Authors:** Raphael Hohlfeld, Sabine Brandt, Anja Bernhardt, Xenia Gorny, Daniel Schindele, Burkhard Jandrig, Martin Schostak, Berend Isermann, Jonathan A. Lindquist, Peter R. Mertens

**Affiliations:** ^1^ Clinic of Nephrology and Hypertension, Diabetes and Endocrinology, Otto-von-Guericke University Magdeburg, Magdeburg, Germany; ^2^ Clinic of Urology, Otto-von-Guericke-University Magdeburg, Magdeburg, Germany; ^3^ Institute of Clinical Chemistry and Pathobiochemistry, Otto-von-Guericke University Magdeburg, Magdeburg, Germany

**Keywords:** clear cell renal cell carcinoma, cold shock proteins, YB-1, DbpA, Akt1

## Abstract

Cold shock proteins are up-regulated by cellular stress and orchestrate inflammatory responses, cell proliferation, and differentiation. Enhanced cold shock protein expression promotes malignant cell transformation; up-regulation is detected in most cancers and associated with poor prognosis. Akt1, a serine/threonine kinase, is a potent oncogene, which activates pro-proliferative and anti-apoptotic signaling pathways, and phosphorylates the cold shock domain. Unexpectedly, chicken-YB-1 abrogates PI3K-Akt-dependent oncogenic cell transformation in embryonic fibroblasts. Here, we addressed the question whether chicken and human Y-box binding protein-1 (YB-1) act differently on cell transformation, and how a related protein, DNA-binding protein-A (DbpA) behaves in comparison. NIH3T3 cells were transduced with lentiviral vectors encoding for myristoylated (constitutive active) Akt1, YB-1, DbpA, or shRNA targeting YB-1 expression. Colony formation assays showed that human YB-1 acts similar to chicken on Akt-dependent cell transformation. This activity was not titratable. Given the correlation of nuclear YB-1 and upregulated DbpA expression in a series of clear cell renal cell carcinomas (*n =* 40) the colony formation assay was extended to include ectopic DbpA expression. DbpA alone prominently induced cell transformation, which was enhanced when constitutive active Akt1 or concomitant YB-1 expression was present. Notably, co-expression of DbpA together with YB-1 abrogated the repressive effect on Akt1 signaling observed with YB-1 alone. Macroscopically, some colonies yielded a remarkable “invasive” phenotype. Thus, cold shock proteins may convey profound anti- and pro-oncogenic effects on Akt-dependent cell transformation. DbpA is able to overcome the anti-oncogenic effects seen with combined YB-1 and Akt signaling in an *in vitro* model of colonial growth.

## INTRODUCTION

Cold shock proteins (CSPs) are among the most evolutionarily conserved proteins. This protein family is characterized by the presence of one or more cold shock domains, which possess nucleic acid binding properties. This endows these proteins with pleiotropic functions, such as the regulation of transcription, translation, and cell proliferation [1].

In humans, the predominant group of cold shock domain proteins is the Y-box family. The prototypic member is Y-box binding protein 1 (YB-1), also known as DNA binding protein B (DbpB), encoded by the gene *Ybx1*. Two additional family members, DNA binding protein A (DbpA) and C (DbpC), are encoded by the genes *Ybx3* and *Ybx2*, respectively.

Besides its nuclear activities as a transcription factor, YB-1 is also localized within the cytoplasm and directs pre-mRNA splicing [2], storage of mRNAs [3, 4], and serves as a key component of messenger ribonucleoprotein particles (mRNPs) [5, 6]. Translational active polysomal mRNPs contain significantly less YB-1 than translational inactive mRNPs [7], thus CSPs determine the translation process. By binding certain internal ribosomal entry sites (IRES), YB-1 is able to specifically enhance translation, e.g. of Snail, Twist, and HIF1α, with relevance for epithelial-mesenchymal transition (EMT) [8–10]. Recent observations confirm that YB-1 is an integral part of the inflammatory response, e.g. following hypoxia [10, 11]. In addition extracellular functions have been described, similar to the high mobility group box protein (HMGB)-1. Secreted upon cytokine stimulation, YB-1 binds extracellular receptor Notch-3 to activate intracellular signaling cascades [12].

The pleiotropic functions of cold shock proteins are intriguing and highly regulated by posttranslational protein modifications and compartmentalization, protein-protein interactions and the interplay of at least three independently acting nuclear localization signals (NLS) and one cytoplasmic retention signal (CRS) [13]. Phosphorylation of serine 102 within the evolutionarily conserved cold shock domain is one of the posttranslational modifications exerted through PI3K/Akt or MAP kinase signaling, events which may lead to nuclear protein translocation [14, 15]. Recent studies have shown that this phosphorylation is not the only condition which mediates nuclear translocation. Phosphorylation of serine 165, likely through casein kinase II (CKII) upon IL1β stimulation [16], or interaction with DACH I also lead to nuclear localization [17].

Elevated cellular YB-1 levels are found in most cancer cells with poor prognosis. Hanahan and Weinberg proposed as “hallmarks of cancer” six biological capabilities that are required for the development of tumors [18, 19]. YB-1 exhibits all these hallmarks of cancer [20]. In human cancers of the breast, prostate, kidney (renal cell carcinoma), skin (malignant melanoma) and colon up-regulated YB-1 expression is a common histological finding, for some cancers DbpA up-regulation has been similarly described. Progressive disease, extensive metastasis formation, shortened survival, and chemotherapy resistance [21–24, 28–30] are associated with high cold shock protein expression in cancers. For example, the aggressive triple-negative (TNBC) breast cancer-subtype shows strong nuclear YB-1 positivity in 70% of the tumors [25]. It was demonstrated, that YB-1 contributes to the conversion of hormone receptor-positive into triple-negative breast cancer cells through chromatin remodeling [26]. On the other hand, silencing of YB-1 expression results in reduced tumor cell growth in TNBC and HER-2 positive breast cancer cells [27].

YB-1 is also involved in a wide range of other pro-carcinogenic pathways. The Ras/ERK/RSK signaling pathway, which is activated by EGF, may propagate YB-1 phosphorylation in colon cancer [28]. Dunn *et al*. demonstrated YB-1 serine 102 phosphorylation by RSK1/2 in breast cancer cells [29]. YB-1 was shown to induce EMT in prostate cancer after EGF induced phosphorylation of serine 102. However, the YB-1-dependent effects on transformed cell phenotypes are not unequivocally demonstrated in all reports, especially for the PI3K/Akt1 signaling pathway. Bader *et al.* demonstrated that overexpression of chicken YB-1 abrogated oncogenetic cell transformation of chicken embryo fibroblasts by the PI3K-Akt signaling-pathway [30]. These results prompted controversies regarding differential effects exerted by chicken and human YB-1. Furthermore, a concentration-dependent anti-oncogenic effect of YB-1 was postulated for melanoma cells. The authors concluded an auto-regulatory loop restricting endogenous YB-1 protein expression [14]. These controversies prompted the present study to clarify whether chicken and human YB-1 act differently on Akt1-dependent cell transformation and how CSP DbpA interferes with cell transformation alone and in concert with Akt1 signaling and YB-1.

## RESULTS

### Effects of human and chicken YB-1 on Akt1-dependent cell transformation in 3T3 fibroblasts

**Figure 1:**
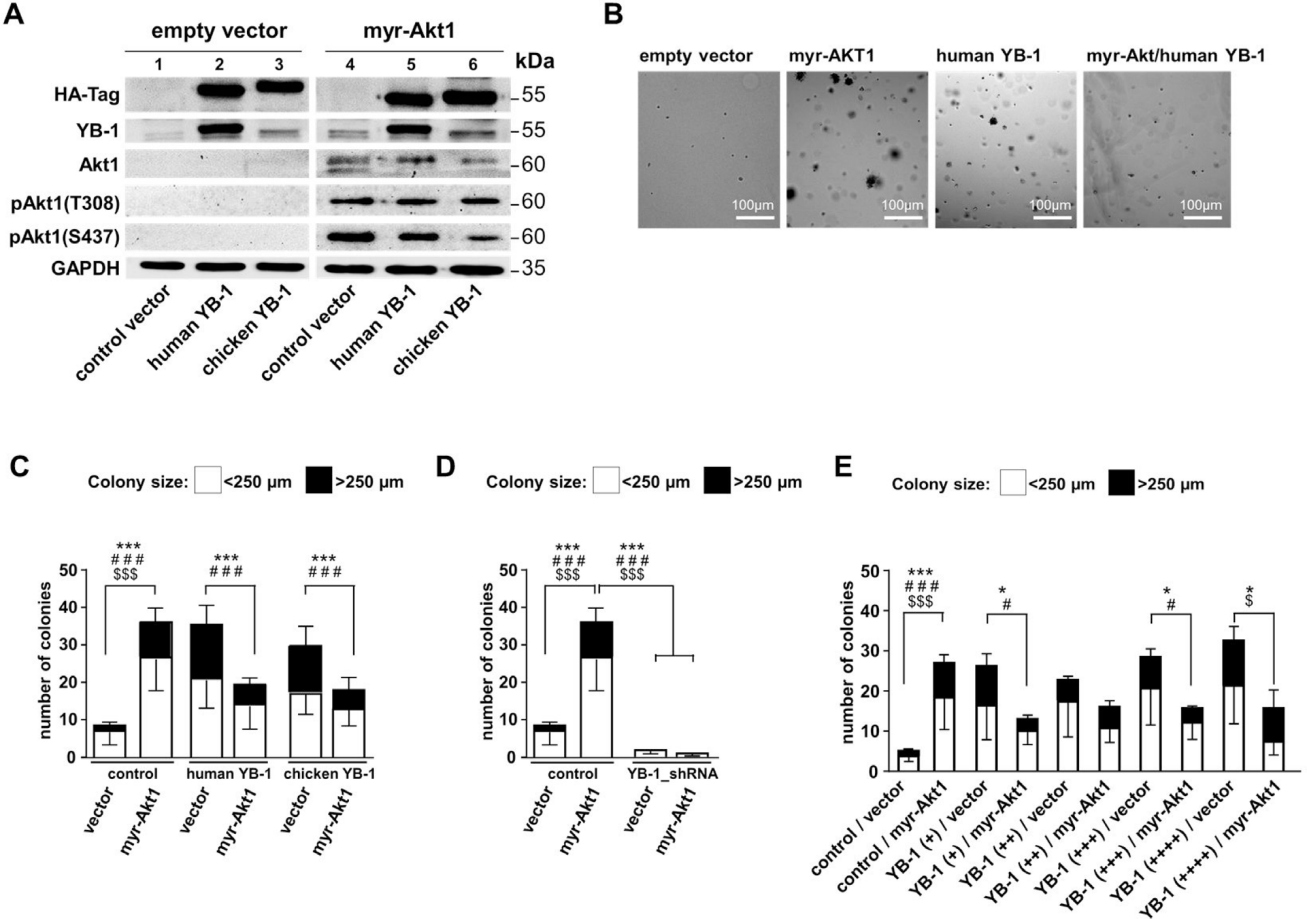
NIH3T3 fibroblast model system to induce Akt signaling and lentivirally overexpress human and chicken YB-1 protein A lentiviral expression system was established to compare functional differences between human and chicken YB-1, especially regarding their oncogenic potential and interference with myr-Akt1 signaling. NIH3T3 cells were lentivirally transduced to enable stable expression of HA-tagged (human)YB-1 and (chicken)YB-1 and myr-Akt1 and YB-1_shRNA. (**A**) Stable protein overexpression was confirmed by Western blotting with indicated antibodies. All samples were run on one gel. (**B**) Soft agar colony forming assays were established with respective overexpression of proteins. (**C**) To assess the oncogenic effects, colony number and sizes (less and more than 250 µm) were recorded as indicators for malignant cell transformation and anchorage independent growth. (**D**) In cells with 80% knockdown of endogenous YB-1 myr-Akt1 had no effect on cell transformation and colony formation was absent. (**E**) In another experimental setup the titration of lentiviral infection was performed to test for concentration-dependent YB-1 effects. However, even at the lowest titer of the expression virus an anti-oncogenic effect was seen with combined expression of myr-Akt1. Statistical analysis is shown for ^*^total number colonies; # large >250 µm colonies; ^$^small <250 µm colonies; ^*/#/$^*p* < 0.05; ^***/###/$$$^*p* < 0.001.

To test the primary question how human (h)YB-1 and chicken (ch)YB-1 influence malignant cell transformation mediated by Akt1 signaling, we first set up experiments similar to Bader *et al.* [30] and tested their ability to propagate or prevent anchorage-independent growth. NIH3T3 cells were lentivirally transduced to enable stable expression of HA-tagged human and chicken YB-1. After three days of culture, a second transduction was initiated to achieve constitutive active Akt1 signaling by introducing myristoylated Akt1 protein (myr-Akt1). For all transduction-steps, control experiments were set up with “empty” lentiviral vectors (Figure 1A). Oncogenic effects were assessed by soft agar colony forming assays (Figure 1B). Colonies were counted and the sizes of colonies determined, which were classified as small (diameter up to 250 µm) and large (diameter more than 250 µm). Respective numbers are summarized for the different conditions in Figure 1C.

Ectopic overexpression of myr-Akt1 or YB-1 led to increased numbers of colonies that also encompassed some with larger diameters. When myr-Akt1 was co-transduced with the expression vector encoding for human YB-1 the number of colonies decreased and were primarily of smaller size, which is in line with the findings for chicken YB-1 reported by Bader *et al.* [6, 30] (Figures 1B and 1C). Thus, although chicken and human YB-1 proteins exhibit considerable differences in their composition within the C-terminal domains the same anti-oncogenic effect is conveyed. For all further experiments vectors encoding for human YB-1 were used.

Additionally, YB-1 knockdown was performed by lentiviral transduction that encoded for YB-1 shRNA (Supplementary Figure 1). Depletion of YB-1 resulted in a substantial decrease of colony formation, which was not overcome by ectopic overexpression of myr-Akt1 (Figure 1D). From these findings one may conclude that Akt1 signaling and cell transformation is dependent on basal endogenous YB-1 expression, however the number of Akt1-transformed cells and cell proliferation is limited in cells with ectopic YB-1 overexpression.

### The anti-oncogenic YB-1 effect on Akt1-dependent cell transformation is not titratable

Next, experiments were designed to determine whether there is a concentration-dependent effect of YB-1 on cell transformation and how this interferes with Akt1-dependent phenotypic changes (Figure 1E). Titrating the ectopic YB-1 expression by increasing the lentiviral vector concentration prompted a gradual increase in colony size and number. In contrast, Akt1-induced colony formation was reduced by ectopic YB-1 expression, as already shown, which was even seen with the lowest concentration of applied lentiviral vector encoding for hYB-1.

### Expression pattern of cold shock protein DbpA and YB-1 in clear cell renal carcinoma

**Figure 2:**
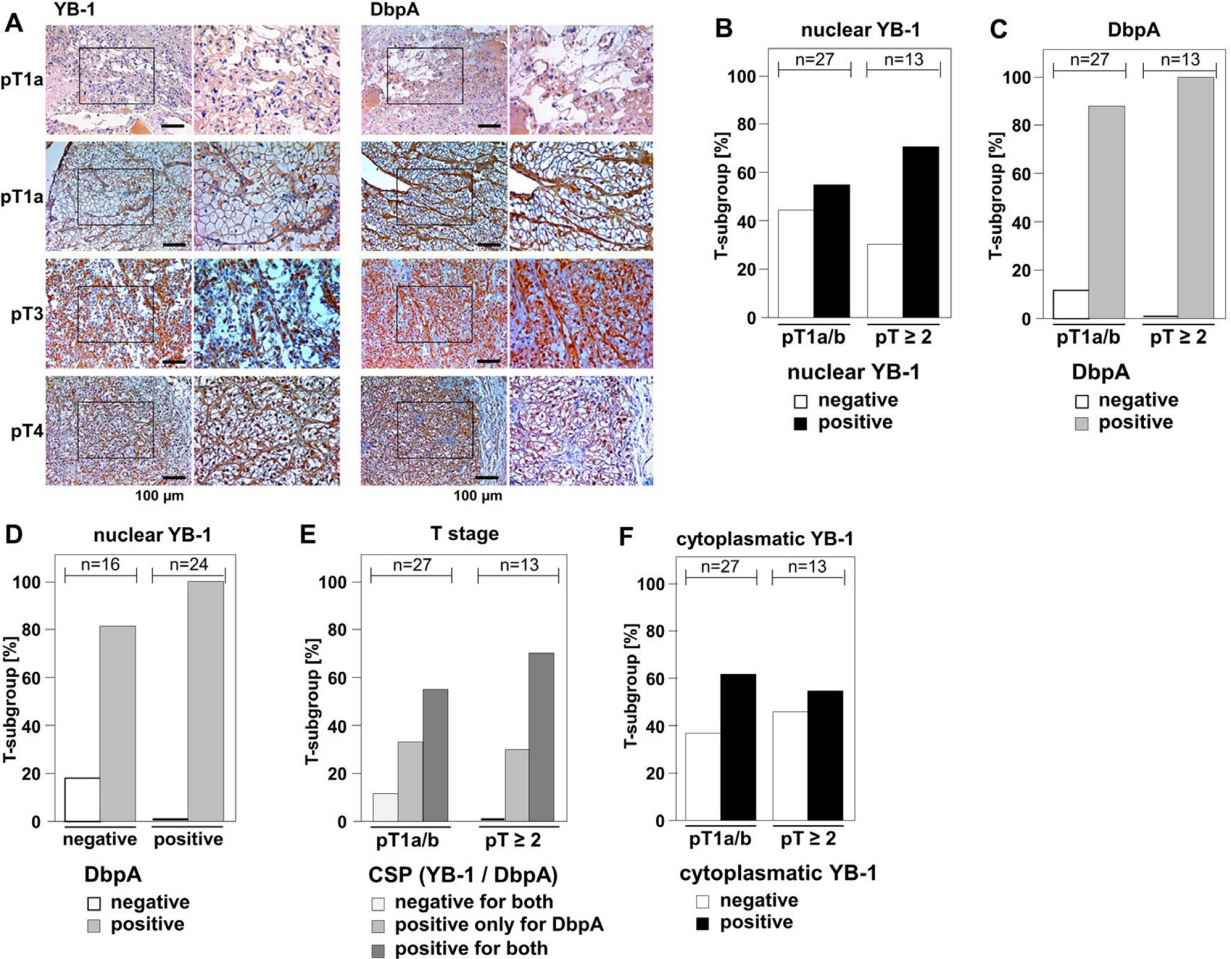
Overexpression of DbpA is associated with overexpression of YB-1 in clear cell renal cell carcinomas (**A**) By means of monoclonal anti-YB-1 and polyclonal anti-DbpA antibodies immunohistochemistry was performed with 40 kidney tissue samples, all diagnosed with clear cell renal carcinomas. Examples of immunohistochemistry are shown for different tumor stages. (**B–F**): Immunodetection of YB-1/DbpA was visually quantified and calculated as present or absent in dependency of tumor stages pT1a/b or pT ≥ 2. DbpA and nuclear YB-1, but not cytoplasmic YB-1, are associated with higher T-stage (**B/C/F**). Absence of DbpA only occurred in YB-1 negative tissues **(D)**. All YB-1 positive tumors exhibited DbpA expression **(D)**. Double positivity for both proteins was highly prevalent in more advanced cancers diagnosed stages pT2 and higher. Double negative tissues were only found in T stage 1 **(E)**.

Few evidence exists regarding the (co-)expression of cold shock proteins in cancer entities, although both proteins, YB-1 and DbpA, have been described to be associated with poor overall outcome when detected at high levels. In clear cell renal cell carcinomas YB-1 expression is associated with an invasive phenotype, however DbpA expression has not been determined for this cancer entity [23, 31]. We set up a pilot study to test for the staining pattern and intensity of YB-1 and DbpA expression determined at the same time in 40 samples. Tissue samples were stained with monoclonal anti-YB-1 [32] and polyclonal anti-DbpA [2] antibodies by immunohistochemistry. Immunodetection of DbpA in the cytoplasm and of YB-1 in the cytoplasm and/or nucleus was seen with these well characterized antibodies that do not cross-react with the respective other cold shock protein. Staining patterns were graded and the main finding was that co-expression of both proteins correlated with advanced tumor stages pT ≥ 2 (Figure 2A).

Nuclear expression of YB-1 was detected in 60% (24/40 positive) of clear cell renal cell carcinomas, whereas DbpA expression was present in 92.5% (*n =* 37/40) of the analyzed tissue samples. Tumors of higher stages (pT ≥ 2) showed a tendency towards higher prevalence of nuclear YB-1 (Figure 2B and Table 1). Absence of DbpA was more likely seen at lower T-stages (pT1a/b; Figure 2C and Supplementary Table 1). All tissue samples that exhibited strong immunopositivity for nuclear YB-1 were also immunopositive for DbpA (Figure 2D). Co-expression of YB-1 and DbpA was associated with higher T-stages, while absence of both proteins was only detected in pT1a/b carcinomas (Figure 2E). Cytoplasmic YB-1 staining did not show a correlation with T-stages (Figure 2F). It has to be emphasized that the limited number of analyzed samples precludes a definite conclusion regarding outcome for “double positive” and “double negative” tissue. However, these results encouraged us to utilize our *in vitro* transformation assay of NIH3T3 cells to test for combined effects of YB-1 and DbpA on colony growth.

**Table 1:** Correlation between nuclear YB-1 and DbpA expression in ccRCC

YB-1	*N*	DbpA		Spearman’s rank correlation coefficient
		neg	pos	
negative	30	3	13	0.349 (*P* = 0.027)
positive	10	0	24	

### Ectopic DbpA expression promotes cell transformation and augments Akt1-dependent anchorage-independent growth in 3T3 fibroblasts

**Figure 3:**
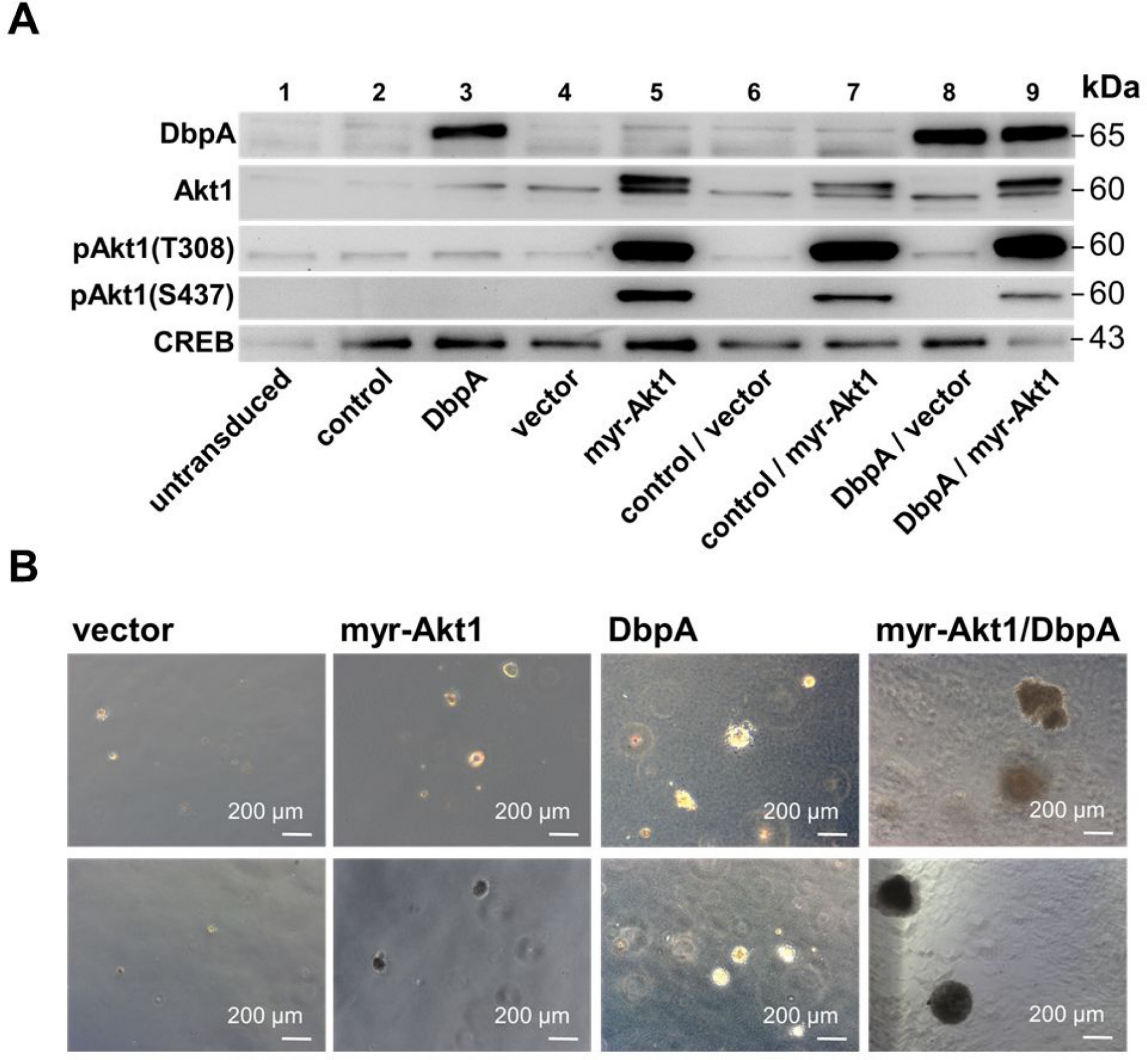
NIH3T3 fibroblast model system for overexpression of myr-Akt1 and DbpA by lentiviral transduction NIH3T3 cells were lentivirally transduced to enable stable expression of human DbpA_A and myr-Akt1. (**A**) Stable protein overexpression was confirmed by Western blotting with indicated antibodies. (**B**) Soft agar colony forming assay was performed to assess the oncogenic potential of expressed proteins alone or in combination. Colony number and sizes (less or more than 250 µm) were assessed. Both, DbpA or Akt1 induced malignant cell transformation. Combined overexpression enhanced the growth potential. Some colonies appeared aggravated in their growth behavior.

**Figure 4:**
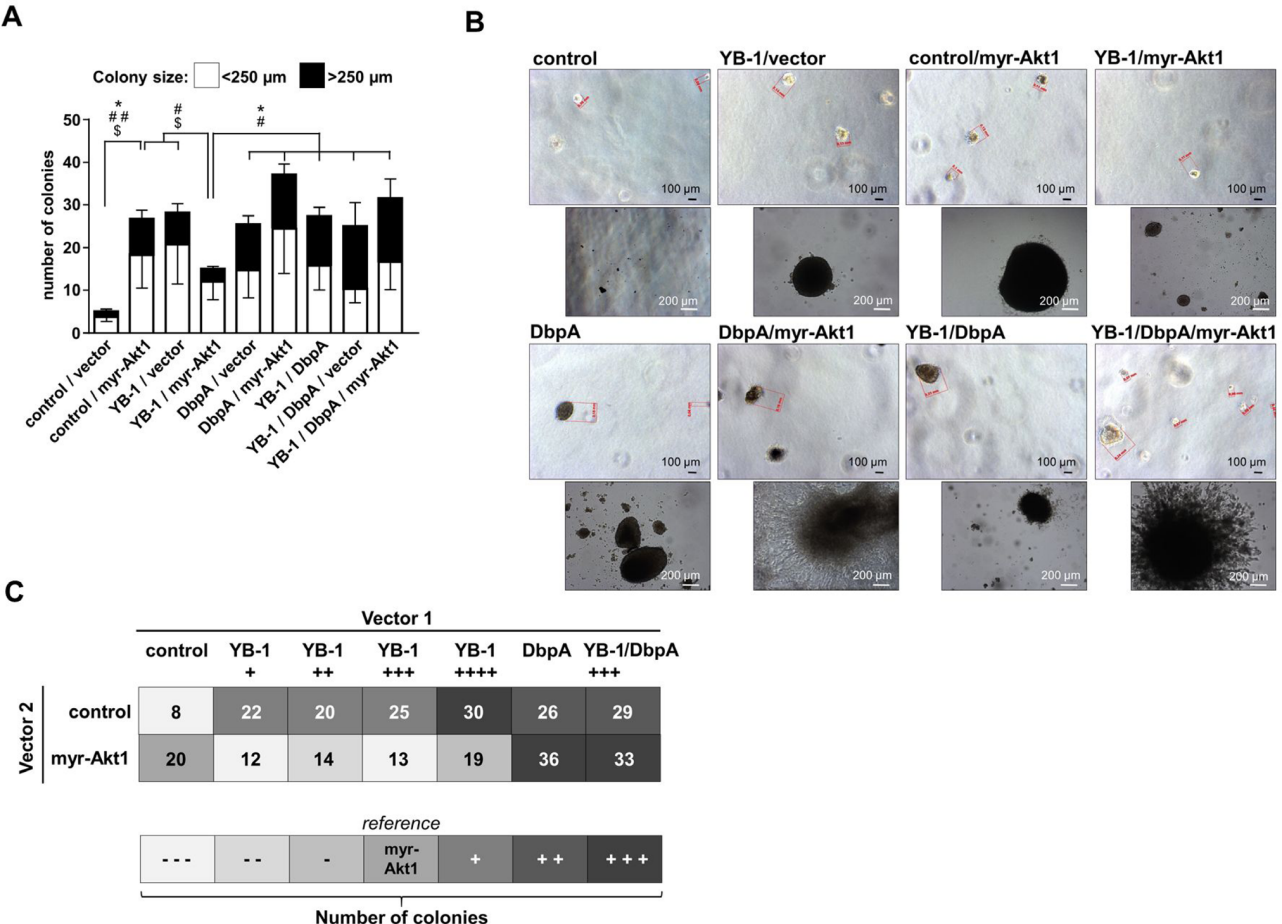
NIH3T3 fibroblast model system for combinatory overexpression of DbpA, YB-1 and myr-Akt1 by lentiviral transduction (**A**) Soft agar colony forming assay was performed to assess the oncogenic potential of ectopically expressed proteins alone or in combination. Colony number and sizes were calculated. Statistical analysis is shown for ^*^ total number colonies; # large >250 µm colonies; $ small <250 µm colonies; ^*/#/$^*p* < 0.05; ^**/##/$$^*p* < 0.01. (**B**) Ectopic expression of DbpA or myr-Akt1 alone or in conjunction with YB-1 resulted in anchorage independent growth of colonies. Some of these colonies with combined myr-Akt1 and DbpA activities exhibited a prominent invasive phenotype with protrusions. (**C**) Combined effects of YB-1, DbpA and myr-Akt1 on oncogenic cell transformation illustrated in a heat map.

To determine the oncogenic properties of DbpA protein and its interference with Akt1 signaling we set up an analogous cell transformation assay as described for YB-1. NIH3T3 cells were lentivirally transduced to achieve ectopic expression of DbpA_A (longer splice variant which includes the alternative domain) in combination or absence of constitutive Akt1 signaling by myristoylated Akt1 (Figure 3A) and quantified colony formation. Expression of DbpA alone resulted in markedly augmented colony formation. In contrast to YB-1, this effect was even more pronounced by co-expression of constitutive active myr-Akt1 (Figures 3B and 4A). Furthermore, co-expression of both proteins, DbpA and YB-1, led to a prominent change of the colony phenotype in some instances with loss of globular appearance and outgrowth of spindle-shaped protrusions into the matrigel.

### Combinatory effects of YB-1, DbpA, and Akt1 signaling on cell transformation

Finally, the matrigel colony formation assay was applied to determine the effects of combined ectopic expression of proteins YB-1 and DbpA on constitutive active myr-Akt1-dependent cell transformation. The combinatorial ectopic expression of YB-1/DbpA, of DbpA/myr-Akt1 as well as YB-1/DbpA/myr-Akt1 all led to a markedly increased colony number, especially of larger sizes (Figure 4A). Notably, ectopic DbpA expression was sufficient to overcome the negative oncogenic interference of YB-1 on Akt1 signaling. YB-1 and DbpA overexpression with or without Akt1 resulted in a strongly increased colony formation. Again some colonies demonstrated a prominent phenotype with extensive protrusions (Figure 4B).

## DISCUSSION

Overexpression of cold shock proteins is associated with most cancers, such as breast cancer [20, 32, 33]. Since protein family members are involved in the regulation of transcription and translation, pre-mRNA splicing, cell cycle progression, DNA repair, nucleo-cytoplasmic cargo of proteins, their repertoire of functions is broad and may be involved in all levels of carcinogenesis. YB-1 was extensively investigated regarding its oncogenic function. Even though its overexpression – especially nuclear localization – is a predominant finding in cancers with poor prognosis, the previous reports by Bader *et al.* [6, 30] describing an anti-oncogenic effect are seemingly contradictory. Taking a closer look at these findings one has to mention that the anti-oncogenic effects described by Bader *et al.* have been obtained with chicken YB-1 in an overexpression system, whereas the findings by Schittek and colleagues [14] are reported for a knock-down of YB-1, which may not prove a direct link of YB-1 with cell transformation *per se*.

The anti-oncogenic effect seems to be associated with two factors: (i) a cytoplasmic localization and (ii) dependency on Akt1 signaling. Schittek and colleagues concluded an auto-regulatory loop restricting endogenous YB-1 protein expression and Bader *et al.* demonstrated inhibition of Akt1-mediated cell transformation by YB-1. First of all, our findings demonstrate the same anti-oncogenic effect for human YB-1. This suggests a non-species-specific anti-oncogenic activity of the evolutionarily conserved YB-1 domains. Neither with chicken YB-1 nor with human YB-1 Akt1-dependent cell transformation was completely blunted, however within the YB-1 concentration range of our ectopic lentiviral-dependent protein expression there was no titration effect. Cell fractionation revealed a predominant cytoplasmic versus nuclear distribution of YB-1 (data not shown). In extension to this *in vitro* study we focused on the combined expression of YB-1 together with the other prominent cold shock protein family member, denoted DNA binding protein-A. Our results indicate that DbpA is a powerful co-factor of YB-1 in carcinogenesis and likely *vice versa* (Figure 4C). Our pilot study on the expression levels of YB-1 and DbpA in clear cell renal cell carcinomas (ccRCC) performed by immunohistochemistry demonstrates that (i) both proteins are mostly upregulated concordantly in tumor cells. (ii) The scenario of combined expression of YB-1 and DbpA at high levels correlates with a higher T-stage. (iii) The DbpA expression seems to be a prerequisite for high YB-1 expression levels, given that all tumor specimen that lacked DbpA protein were also immuno-negative for YB-1. Conversely, there were some samples with DbpA protein expression only. From the literature, a similar finding was reported on DbpA expression correlating with poor outcome in hepatocellular carcinoma [34], again pinpointing to the inferior relevance of YB-1 compared to DbpA for cell transformation. We for the first time demonstrate that ectopic DbpA overexpression transforms cells *in vitro*. This may be explained by the homology within the cold shock domain, which may confer the respective effects.

Due to our finding that DbpA was more often present in ccRCC than YB-1 and that the pro-oncogenic effect of DbpA – alone or in concert with myr-Akt1 was stronger than the effect of YB-1 and that DbpA was able to overcome the anti-oncogenic interference of YB-1 and myr-Akt1, we conclude that DbpA is a powerful oncogene.

From our findings, we conclude, that carcinogenesis – or at least progress in malignancy – requires the overexpression of both YB-1 and DbpA. Direct interaction of both proteins was already demonstrated [23, 35]. The functional relevance of this interaction is not well investigated.

We also adopted the converse approach with knockdown of YB-1 protein. As result, a significantly impaired anchorage-independent growth was seen, an effect reported before [31]. Myr-Akt1 overexpression was not able to overcome this proliferation impairment, suggesting that YB-1 acts upstream of Akt1 in cell-cycle regulation.

Our *in vitro* findings are compelling; however, the immunohistochemistry data are rather preliminary and need confirmation. It will be of interest to further dissect the mechanistic activities of DbpA for cell phenotypes, e.g. in cells of different provenience. It is remarkable that DbpA expression is restricted to smooth muscle cells in healthy tissue and tightly upregulated in myofibroblasts under inflammatory conditions [2]. Thus, the expression pattern is highly regulated. Knockout animals reveal no apparent phenotype in the absence of disease [36]. From these results one may conclude that DbpA has no effect on cell proliferation *per se*. Yet, extensive analyses with knockout of DbpA revealed that mitogenic effects exerted by platelet-derived growth factor-B are blunted in the absence of DbpA [2]. The combined effects may merit closer inspection and evaluation of CSP expression levels with the quest of establishing prognostic markers and therapeutic targets. Of note, most of the signaling pathways related to aberrant signaling in cancer cells target residues within the cold shock domain which is identical between DbpA and YB-1 [20, 36]. Thus joint forces may act on both cold shock proteins and render phenotypic changes of cells that exhibit an invasive growth behavior.

## MATERIALS AND METHODS

### Cell cultures

The HEK 293T cell line “LentiX” (Clontech) was cultured in high glucose-containing Dulbecco’s modified Eagle’s medium (DMEM; Gibco) supplemented with 10% fetal calf serum (FCS, PAN Biotech) and 100 U/ml penicillin, 100 μg/ml streptomycin (Gibco) at 37° C at 5% CO_2_, unless otherwise stated. Mouse NIH3T3 cells was cultured in high glucose-containing Dulbecco’s modified Eagle’s medium (DMEM; Gibco) supplemented with 10% fetal calf serum (FCS, PAN Biotech) and 100 U/ml penicillin, 100 μg/ml streptomycin (Gibco).

### Plasmids and plasmid construction

The lentiviral transfer plasmids pCCL-EF1α-Dest:SCP-eGFP and pCCL-EF1α-Cherry:SCP-eGFP were kindly provided by Stephan Geley (Department for Molecular Pathophysiology, Medical University Innsbruck). Chicken and human YB-1 with C-terminal HA-tag and human DbpA_A were synthesized by Genscript and cloned into pDREAM-MCS. Constructs were transferred from pDREAM into pCCL-EF1α-Dest:SCP-eGFP by Gateway cloning. pCDH-puro-myr-HA-Akt1 was a gift from Jialiang Wang (Department of Neurological Surgery, Vanderbilt University Medical Center, Nashville) [37].

### Lentivirus production, titer determination and transduction

Lentiviral production and determination of titers was performed as previous described with minor modifications [2, 30]. 8 × 10^5^ cells per 6-well or 3 × 10^6^ cells per 10 cm dish (HEK293T LentiX) were seeded the day before viral transduction. Transduction was performed by means of calcium phosphate precipitates. Chloroquine (25 µM) was added to the cells immediately before the transfection. 16 h post viral transduction the medium was replaced with DMEM supplemented with 10% FCS, 100 U/ml penicillin, 100 μg/ml streptomycin, 10 mM HEPES buffer (Gibco), 1 mM sodium pyruvate (Gibco), 1× non-essential amino acids (Gibco) and 1mM sodium butyrate (Sigma). Virus containing media was collected after 24 hours, sterile filtered through a 45 µm filter and stored at 4° C. New medium was added to the producer cells. A second collection was made after a further 24 hours. Viruses were concentrated by centrifugation at 35,000 × g, 4° C for 90 minutes. The virus pellet was resuspended in PBS or Opti-MEM media. Concentrated virus stocks were either used directly or stored at –80° C. For transduction, media of target cells was replaced by fresh culture medium containing 4 µg/ml polybrene (Sigma). Concentrated virus was added and cells were incubated for 6 hours [30].

### Soft agar colony formation assay

Anchorage-independent growth, as an indicator for malignant cell transformation, was assayed by the agarose soft agar transformation assay. After lentiviral transduction, cells were trypsinized and suspended in growth medium. Solutions of 1.33× DMEM and 1.4% agarose (low gelling temp., cell culture grade; Sigma) were mixed with 7.5 × 10³ cells and plated out in 12-well dishes. After gelling of the 1xDMEM/0.35% agarose cell suspension at room temperature, cultures were kept at 37° C under 5% CO_2_ and 95% H_2_O saturation for 3–4 weeks. All colonies in the optical axis of four medium fields, one in each quadrant of the well, were count and sizes were determined optically.

### Cell lysate preparation and Western blot analysis

Cells were lysed in RIPA buffer (50 mM Tris-HCl, 150 mM Nonidet P-40, 1 mM sodium deoxycholate, 1 mM EDTA, and 1 mM sodium orthovanadate) containing complete protease inhibitor cocktail (Roche) and Phosphostop phosphatase inhibitor (Roche) at 4° C for 30 min as decribed.

Protein concentrations were determined by protein assay (Bio-Rad). Denatured protein samples were separated using 12% SDS-PAGE and blotted onto nitrocellulose membranes. Membranes were blocked with 5% dry milk in TBS/Tween and incubated with primary antibodies diluted in TTBS overnight at 4° C. Primary antibodies included YB-1 c-term (polyclonal rabbit; 1:1000; Mertens/Eurogentec); DbpA (polyclonal rabbit; 1:1000; Mertens/Eurogentec); Akt1 (monoclonal mouse; 1:1000; Cell Signalling #2967); pAkt(S473) (monoclonal rabbit; 1:1000; Cell Signalling #4058); pAkt(Thr308) (monoclonal rabbit; 1:1000; Cell Signalling #2965); HA-Tag (polyclonal rabbit, 1:1000; Santa Cruz #sc-805). Secondary HRP-conjugated goat anti-rabbit IgG (Biozol) or goat anti-mouse IgG (Biozol) antibodies were added for 60 minutes. Following additional washing steps SuperSignal chemiluminescence substrate (GE Healthcare; #RPN2232) was added and emitted light detected by Intas imaging system.

### Immunohistochemistry

Formalin-fixed paraffin-embedded tissue samples were deparaffinized and rehydrated. Antigen-retrieval was done by boiling in 10 mM sodium citrate buffer (pH 6.0) for 10 min. Tissue sections were blocked with PBS containing FCS 20% and Tween 0.1% for 1 hour, followed by over night incubation with primary antibodies dissolved in PBS/FCS 1%/Tween 0.1% at 4° C in a humidified chamber overnight. After washing the sections were incubated with HRP labeled secondary antibodies or HRP-labeled streptavidin, diluted in 0.1% Tween-PBS for 1 hour at room temperature. The staining was achieved by incubation in diaminobenzidine (DAB) for 90 to 120 seconds, followed by two washing steps of 5 minutes in dI H_2_O and counterstaining with hematoxylin.

### Patients and tissue specimens

This study included 40 patients who underwent radical nephrectomy for renal cell carcinoma at the Department of Urology, Otto-von-Guericke University Magdeburg. All patients provided informed written consent prior to surgery in compliance with the Declaration of Helsinki and the approved protocols of the medical ethics committee of the Otto-von-Guericke University Magdeburg (# 87/11). The median age at surgery was 66 years. pT1/2 stages predominated (*n* = 30/40). The majority of patients had no lymph node or vena cava invasion. Tissue samples were selected with a male:female ratio of 1:1 in order to avoid gender bias. Detailed information on patient characteristics is described in Table 2.

**Table 2:** Characteristics of ccRCC patients

Characteristics	Variables	Cases	%
Patients with ccRCC^*^		40	100
Gender			
	female	20	50
	male	20	50
Age at surgery (years)			
	Median	65.7	
	range	38–78	
Max tumor size (cm)			
	Median	4.0	
	range	1.4–14	
Stage			
	T1a,b	27	67.5
	T2a,b	3	7.5
	T3a,b	7	17.5
	T4	3	7.5
Fuhrman grade			
	G1	18	45
	G2	16	40
	G3	3	7.5
	G4	3	7.5
Lymph node metastases			
	Nx	32	80
	N0	7	17.5
	N1	0	0
	N2	1	2.5
Distant metastases (cM)			
	Mx	32	80
	M0	4	10
	M1	4	10
Lymph vessel invasion			
	No	37	92.5
	Yes	3	7.5
Renal vein invasion			
	No	32	80
	Yes	8	20
Resection status			
	0	38	95
	1	2	5

^*^ccRCC clear cell renal cell carcinoma.

### Statistical analysis

The pair-wise comparison of the data was performed using the Student’s *t*-test (GraphPad Prism) for the total number of colonies (^*^) as well as among large (^#^) and small (^$^) colonies; ^*^p < 0.05, ^**^p < 0.01, and ^***^p < 0.001.

## SUPPLEMENTARY MATERIALS FIGURE AND TABLE


