# Supplementary material for: Crosstalk between Akt signaling and cold shock proteins in mediating invasive cell phenotypes
Source: Oncotarget. 2018 Apr 10;9(27):19039–49. doi: 10.18632/oncotarget.24886 (PMC5922376; doi:10.18632/oncotarget.24886)
Supplement: Supplementary file 1 [file oncotarget-09-19039-s001.pdf]

Crosstalk between Akt signaling and cold shock proteins in mediating invasive cell phenotypes

SUPPLEMENTARY MATERIALS

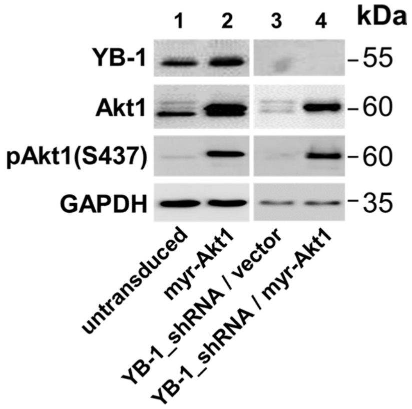

Supplementary Figure 1: Confirmation of lentiviral myr-Akt1 overexpression and YB-1 knockdown. The overexpression of lentiviral myr-Akt1 and knockdown of YB-1 were confirmed by Western blotting using the indicated antibodies. GAPDH is included to control for equal loading.

Supplementary Table 1: Expression of nuclear YB-1 and DbpA in clear cell renal cell carcinoma

Parameter		*N*	YB-1 expression nuclear		*P* Value	DbpA expression		*P* Value
			–	+		–	+	
T stage	pT1a/b	27	12	15	0.318	3	24	0.296
	pT ≥ 2	13	4	9		0	13	

Parameter		*N*	YB-1/DbpA expression double negative		*P* Value	YB-1/DbpA expression double positive		*P* Value
			**yes**	**no**		**no**	**yes**	
T stage	pT1a/b	27	3	15	0.296	12	15	0.318
	pT ≥ 2	13	0	13		4	9	
